# Burst Firing in a Motion-Sensitive Neural Pathway Correlates with Expansion Properties of Looming Objects that Evoke Avoidance Behaviors

**DOI:** 10.3389/fnint.2015.00060

**Published:** 2015-12-14

**Authors:** Glyn A. McMillan, John R. Gray

**Affiliations:** Department of Biology, University of SaskatchewanSaskatoon, SK, Canada

**Keywords:** sensory coding, bursting, vision, locust, neuron, DCMD

## Abstract

The locust visual system contains a well-defined motion-sensitive pathway that transfers visual input to motor centers involved in predator evasion and collision avoidance. One interneuron in this pathway, the descending contralateral movement detector (DCMD), is typically described as using rate coding; edge expansion of approaching objects causes an increased rate of neuronal firing that peaks after a certain retinal threshold angle is exceeded. However, evidence of intrinsic DCMD bursting properties combined with observable oscillations in mean firing rates and tight clustering of spikes in raw traces, suggest that bursting may be important for motion detection. Sensory neuron bursting provides important timing information about dynamic stimuli in many model systems, yet no studies have rigorously investigated if bursting occurs in the locust DCMD during object approach. We presented repetitions of 30 looming stimuli known to generate behavioral responses to each of 20 locusts in order to identify and quantify putative bursting activity in the DCMD. Overall, we found a bimodal distribution of inter-spike intervals (ISI) with peaks of more frequent and shorter ISIs occurring from 1–8 ms and longer less frequent ISIs occurring from 40–50 ms. Subsequent analysis identified bursts and isolated single spikes from the responses. Bursting frequency increased in the latter phase of an approach and peaked at the time of collision, while isolated spiking was predominant during the beginning of stimulus approach. We also found that the majority of inter-burst intervals (IBIs) occurred at 40–50 ms (or 20–25 bursts/s). Bursting also occurred across varied stimulus parameters and suggests that burst timing may be a key component of looming detection. Our findings suggest that the DCMD uses two modes of coding to transmit information about looming stimuli and that these modes change dynamically with a changing stimulus at a behaviorally-relevant time.

## Introduction

Representation of salient sensory information within neuronal spike trains is a complex process that can involve rate coding, averaging across time, and time coding that relies on precise timing of events such as individual spikes or bursts of high frequency spikes. Rate coding implies that information is averaged over a period of time and that variability in individual spike timing represents random neural noise (Stein et al., 2005). However, growing evidence suggests that rate coding may be important for low frequency components of a stimulus and that time coding can account for rapid fluctuations in stimulus properties, which may be more reflective of natural sensory signals. Time coding, may also be important for the flow of neural information through circuits that drive behavior (Salinas and Sejnowski, 2001). Moreover, spike times and firing rates may be used independently to represent different aspects of a stimulus variable (VanRullen et al., 2005), are not mutually exclusive, and can be used in the same sensory neurons (Stein et al., 2005). If information provided by sensory neurons is effectively transmitted within a certain time window, the timing of those spikes may also be important in gating processes that control information flow (Salinas and Sejnowski, 2001).

The image of a rapidly approaching (looming) object is an evocative visual stimulus, eliciting avoidance reactions in many animal species (Maier et al., 2004; Santer et al., 2005; Wu et al., 2005; Oliva et al., 2007) and neurons responsible for the detection and relay of looming stimuli are present in birds (Sun and Frost, 1998) and many insects (Simmons and Rind, 1992; Hatsopoulos et al., 1995; Wicklein and Strausfeld, 2000). Looming stimuli also trigger the production of adaptive behavioral responses in locusts (Simmons and Rind, 1992; Judge and Rind, 1997; Gabbiani et al., 1999; Gray et al., 2001; Simmons et al., 2010; McMillan et al., 2013). One of the most widely studied neural pathways involved in avoidance behaviors consists of an identified motion-sensitive neuron, the lobula giant movement detector (LGMD) and its post synaptic partner, the descending contralateral movement detector (DCMD). The DCMD excites thoracic interneurons and motor neurons involved in flight steering and jumping (Burrows and Rowell, 1973; Matheson et al., 2004) and may gate information into the flight rhythm to modify the course during flight or initiate a glide (Reichert and Rowell, 1985, 1986; Reichert et al., 1985; Santer et al., 2006; Simmons et al., 2010). The DCMD response has also been linked to specific phases in a jump (Fotowat et al., 2011). Although sensitive to many other forms of object motion (Guest and Gray, 2006; McMillan and Gray, 2012; Dick and Gray, 2014; Silva et al., 2015), the LGMD/DCMD pathway responds preferentially to looming (Schlotterer, 1977; Rind and Simmons, 1992; Hatsopoulos et al., 1995; Judge and Rind, 1997; Gabbiani et al., 1999, 2001, 2002; Matheson et al., 2004).

**Figure 1:**
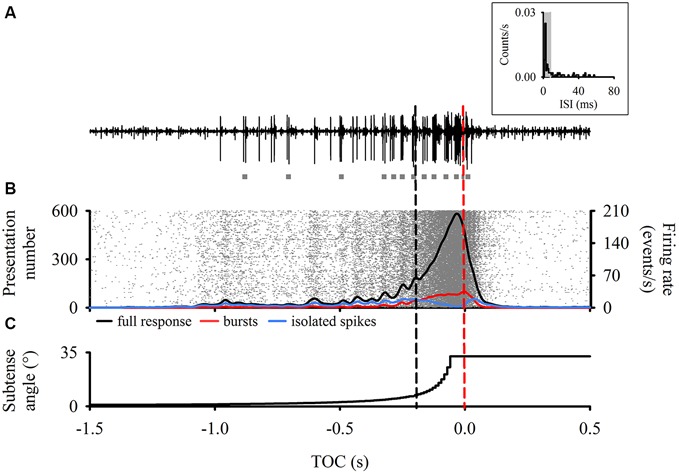
**Descending contralateral movement detector (DCMD) responses time aligned to the perceived time of collision (TOC, red dashed line) of a laterally looming visual stimulus. (A)** Raw extracellular neuronal recording representing a typical DCMD response (large spikes) to our looming stimulus. Note the presence of multiple bursts (gray squares) throughout the recording. Inset—Inter-spike Intervals (ISI) histogram from a single presentation to one animal highlighting that most of the ISIs are within 8 ms (shaded area), which defines spikes within bursts (Quantification of a burst is described in methods). **(B)** Raster plot (*n* = 600 responses) with overlay of a 50 ms Gaussian smoothed (1 ms bin width) mean full DCMD firing rate (black line), burst firing rate (red line) and isolated single spike firing rate (blue line). Each row of rasters represents the response of a single DCMD neuron and each raster (in gray) represents a single spike (N = 20 animals with 30 presentations to each). Rasters were organized in ascending order based on the timing of the spike before TOC. Note the consistent vertical banding pattern in the raster plot. Also note that the more distinctive oscillations in the mean response end around 200 ms before TOC (black dashed line). Note the decline in single spike firing rate after *t* = 200 ms, where bursting dominates and peaks around TOC. **(C)** Vertical step line plot representing the change in subtense angle of the looming stimulus. As the edges of the virtual disc expanded, the full DCMD response in **(B)** increased to a peak that occurred before TOC.

**Figure 2:**
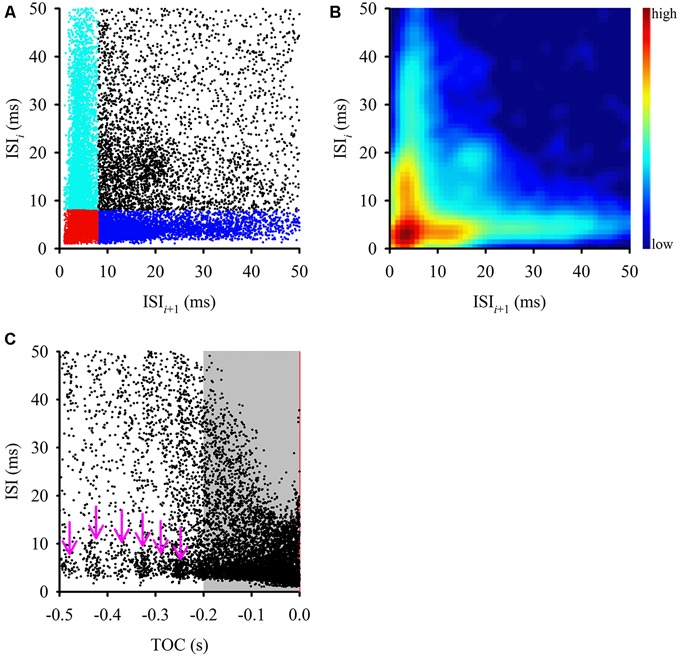
**Comparison of interspike intervals (ISIs) during the entire stimulus. (A)** This ISI return map compares one ISI (*y*-axis) with the following ISI (*x*-axis) and displays a fan-shaped distribution of ISIs with a tight cluster of points at the origin (in red) and two other clusters along the axes (cyan and blue). Clustering along each axis was defined by an 8 ms ISI within a burst. This type of distribution is typical of bursting neurons, where a cluster along the *y*-axis (cyan) represents first ISIs of each burst, a cluster along the *x*-axis (blue) represents the ISIs that follow bursts, the cluster in the bottom left corner (red) represents intra-burst ISI and the more scattered clusters (black) represents ISIs between isolated spikes. **(B)** Joint ISI distribution heat map showing local density of clusters (smoothed with a 3 ms Gaussian radius) and highlighting the clusters identified in **(A)**. The color scale to the right of the graph defines relative local densities. **(C)** ISIs tended to shorten as the stimulus reached TOC (red vertical line), however there was a relatively constant ISI distribution in short intervals along the *x*-axis (pink arrows). During the final 200 ms before TOC, the ISIs became progressively shorter, which mirrors the mean DCMD plot in Figure 1B.

**Figure 3:**
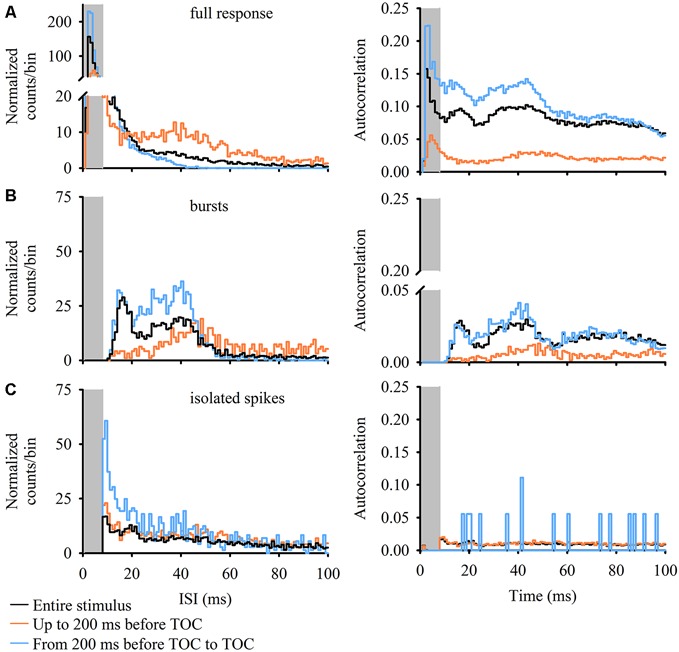
**ISI histograms (ISIh; left panels) and autocorrelations (right panels) of DCMD responses for full responses (A) DCMD bursts (B) and isolated spikes (C).** Data for ISIh (counts/bin) were normalized to the number of interspike intervals within the train of events (bursts or spikes, see “Materials and Methods” Section). While partially masked within the full DCMD response in all stimulus epochs **(A)** there is a bimodal distribution of burst intervals **(B)** in the full DCMD response (black line) and in bursts during the final 200 ms before TOC (blue line); one with shorter ISIs and a second with longer ISIs around 40 ms. This trend is more clearly visualized in the DCMD’s 100-ms autocorrelation. Following identification of bursts, we found a relatively unimodal distribution with highest rates occurring around 40 ms; this trend was also reflected in the associated autocorrelations. Overall, there was no clear trend in the distribution of isolated spikes.

Several pieces of evidence provide a compelling rationale for exploring putative DCMD bursting (brief episodes of high frequency firing) in response to looming stimuli. First, we observed burst-like activity from many studies focused on responses to looming (for example, Figure 1E, Rind, 1996; Figure 2, Gabbiani et al., 1999; Figure 1A, Gabbiani et al., 2001; Figure 2, Guest and Gray, 2006; Figure 1B, Money et al., 2006, Figure 1D; Santer et al., 2006; Figure 3, Rogers et al., 2010; Figure 1, Fotowat et al., 2011; Figure 6C, Jones and Gabbiani, 2012; Figure 2A, McMillan and Gray, 2012; Figure 2, Silva et al., 2015). Second, various studies propose that DCMD shows intrinsic bursting properties. Gabbiani and Krapp (2006) describe the LGMD (the DCMD’s presynaptic input O’Shea and Williams, 1974) as “an intrinsically bursting neuron” while other reports indicated that the DCMD discharges a high-frequency series of bursts in response to small moving objects (Pearson and O’Shea, 1984; Santer et al., 2005; Rogers et al., 2010). Using depolarizing current pulses above threshold, the LGMD response showed a bimodal interspike interval (ISI), supporting a bursting behavior (Gabbiani and Krapp, 2006).

Bursting neurons occur in many sensory systems, including mammalian auditory (Eggermount and Smith, 1996) and visual (Sherman, 2001) systems, weakly electric fishes (Krahe and Gabbiani, 2004), and auditory neurons in insects, such as crickets (Marsat and Pollack, 2006) and locusts (Eyherabide et al., 2008). Information within a burst signal can be more precise compared to a single action potential and may reliably transmit a signal from one neuron to another, avoiding synaptic transmission failure (Chen et al., 2009). Thus, bursts can be important in signaling the occurrence of behaviorally relevant salient sensory cues (Hildebrandt et al., 2015). For example, bursts in pyramidal cells in the electro-sensory lateral line lobe of weakly electric fish are associated with electric field distortions caused by moving prey (Oswald et al., 2004). It has also been shown that bursts reliably predict behavioral responses. Marsat and Pollack (2006) demonstrated that burst activity in an identified ultrasound-sensitive auditory interneuron (AN2) of crickets signals stimulus features of echo-locating bats. Crickets respond to ultrasound stimuli with avoidance responses that are triggered by AN2 (Nolen and Hoy, 1984). Marsat and Pollack (2006) proposed that isolated spikes in the AN2 neuron may play a role in encoding cricket song and contributes to behavioral responses to these stimuli, while bursting may be used when avoiding bats, given that only bursts elicit strong behavioral responses. Thus, multiple modes of encoding in single neurons may have context-dependent behavioral implications.

The DCMD and other collision detecting neurons have been described to use a rate code to signal an imminent collision. However, it was also assumed that cricket auditory neurons detected bat calls using a rate code (Nolen and Hoy, 1984), but later shown that bursting is the reliable code (Marsat and Pollack, 2006). Indeed, within the time frame of a single wing beat, the deviation neuron in the locust, DNI, uses the timing of spikes to reliably transmit information regarding changes in pitch (Simmons and van Steveninck, 2010), which presumably aids course deviation correction during flight by responding to changes in the horizon. Within the DCMD spike train there may exist timing information that plays a pivotal role in the control of rhythmical motor output.

Here, we identified and quantified bursting patterns in DCMD responses to looming. Similar to other sensory neurons (e.g., the AN2 in crickets; Marsat and Pollack, 2006), the DCMD may use two modes of coding to transmit sensory stimuli information. While AN2 is an auditory descending neuron and the DCMD a visual descending neuron, both are known to trigger avoidance responses. We also re-analyzed data from a previous experiment (Dick and Gray, 2014) and applied the bursting algorithm described here to determine if bursting occurs across a range of stimulus parameters.

## Materials and Methods

### Animals

Twenty adult male *Locusta migratoria* were used for experimentation. Locusts were selected at least 3 weeks past their imaginal molt obtained from a crowded colony maintained in the Department of Biology at the University of Saskatchewan (25–28°C, 12 h:12 h light:dark). Experiments were carried out during early to late afternoon at room temperature (~25°C).

### Animal Preparation

After removing the legs and clipping the wings, a rigid tether was attached to the ventral surface of the thorax using 3M^TM^ Vetbond^TM^ Tissue Adhesive 1469SB (3M Animal Care Products, St. Paul, MN, USA). A small patch of ventral cervical cuticle was removed to expose the underlying paired connectives of the ventral nerve cord anterior to the prothoracic ganglia. The exposed tissue was bathed in a drop of locust saline (147 mmol NaCl, 10 mmol KCl, 4 mmol CaCl_2_, 3 mmol NaOH, 10 mmol Hepes, pH 7.2) and the preparation was transferred to the recording stage. Neuronal recordings were obtained from the left ventral nerve connective using a bipolar silver wire hook electrode insulated with a mixture of Vaseline and mineral oil and a copper ground electrode was inserted into the abdomen. The entire preparation was then rotated so that the locust was oriented dorsal-side up with its longitudinal axes 12 cm away and perpendicular to the apex of the rear projection dome screen and the right eye was aligned with the azimuthal and elevation axes of the dome apex (see Figure 1 of Guest and Gray, 2006). In this orientation 0° was directly in front of the locust, 180° was directly behind, and 90° was aligned with the center of the eye. The preparation was left for 30 min in front of a projected white visual field (background luminance = 430 cd/m^2^) before the experiment started to allow the animal to acclimate to the experimental setup. To prevent confounding effects of neural habituation, the interval between each presentation was at least 3 min.

### Visual Stimulation

The procedure used for visual stimulus generation and data acquisition was similar to that used previously (Guest and Gray, 2006; McMillan and Gray, 2012; Dick and Gray, 2014; Silva et al., 2015). Visual stimuli were created using the Vision Egg visual stimulus generation software (Straw, 2008) on a Python programming platform and represented as 1024 × 1024 pixel portable network graphics (png) files. Individual pixel sizes on the projection screen were approximately 0.7 mm, corresponding to a visual subtense angle (*θ*) of ~0.4°, which is below the 1° resolution of individual ommatidia (Horridge, 1978). The ratio of the half size of a symmetrical object (*l*) and the absolute velocity (|*v*|) may be used to calculate a single value that relates to the increase in angular subtense during a looming approach (see Gabbiani et al., 1999). The visual stimulus, a 7 cm diameter black disc traveling 3 m/s (*l*/|*v|* = 12 ms), was scaled in real-time at 85 frames/s, which is above the flicker fusion frequency of the locust eye (Miall, 1978), and projected onto a specialized rear projection dome screen using a Sony VPL-PX11 LCD data projector. An approach velocity of 3 m/s matches the average flight speed of a locust (Baker et al., 1981) and has been used in many previous studies on DCMD responses to looming objects. To account for the distortion due to projection onto the curved surface of the screen, correction factors were embedded in the Vision Egg code. A 1.2 ms transistor-transistor logic (TTL) pulse included in each video frame and the vertical refresh synchronization pulse (vsync) from the video card (NVIDIA GeForce4 Ti4200 128 MB) were used to align physiological recordings with events associated with the stimuli. The final frame of each presentation was determined using the last TTL pulse, which indicated when the object had disappeared from the screen. The corresponding vsync pulse determined the start time of the rendering of this frame. The luminance values and Michelson contrast ratio (0.48) were similar to those used previously (Guest and Gray, 2006; McMillan and Gray, 2012; Dick and Gray, 2014; Silva et al., 2015). Twenty animals were presented with 30 identical looming stimuli that approached the right eye at 3 m/s and 90° in the azimuthal plane. Each approach started 400 cm away (*θ* = 1°) and stopped 12 cm away from the eye (*θ* = 32.5°).

### Data Acquisition

For each presentation, neuronal activity from the left cervical connective and stimulus pulses were recorded continuously and stored for offline analysis. Recorded activity was amplified with a differential AC amplifier (A-M Systems, model No. 1700, gain = 10,000) and sampled at 25 kHz. An RP2.1 enhanced real-time processor (Tucker-Davis Technologies, Alachua, FL, USA) with Butterworth filter settings of 100 Hz (high-pass) and 5 kHz (low-pass) was used to store the data. Relatively large DCMD spikes were isolated from lower amplitude nerve cord activity using threshold and manual discrimination settings in Off-line Sorter (Plexon, Dallas, TX, USA; Figure 1A). DCMD spike times and stimulus events were exported to Neuroexplorer spike train analysis software (NEX Technologies, Littleton, MA, USA) for analysis.

### Burst Analysis

Predetermined burst detection analysis is difficult, due to the wide variety of burst structures and patterns, compounded by the challenge of the context in which a burst is produced (i.e., stimulus environment). However, examination of the ISI distribution of the response to a stimulus will usually reveal if bursting is present (Chen et al., 2009). Ideally, an ISI histogram will reveal a burst firing pattern of the neuron if only two groups of intervals are present: a combination of short intervals (intra-burst intervals) and long intervals (inter-burst intervals, IBIs; Cocatre-Zilgien and Delcomyn, 1992). The ISI (Poincare) return plot displays the current ISI against the next ISI in a spike train and provides insight into the second order statistics of the ISI distribution and also aids in burst identification based on similar criteria as the ISI histogram (see Figure 2 for description).

Burst analysis was completed in Neuroexplorer using parameters that were determined by values used previously and by our own data. We quantified a burst event by having at least two successive spikes occurring at a minimum duration of 2 ms (Marsat and Pollack, 2006). Normalized ISI histograms (ISIh) were plotted for all stimulus epochs (described below) and for full DCMD responses, bursts and isolated spikes. Since there were different numbers of events (spikes or bursts) for data within each stimulus epoch, and thus different numbers of interspike intervals for each train, the data (counts/bin) were normalized to the number of interspike intervals. This allowed us to better identify trends across different stimulus epochs and event types. An examination of our ISIh revealed that most intervals were below 8 ms (see Figure 1A (inset) and “Results” Section). Therefore, we treated ISIs ≤ 8 ms as spikes within a burst. Subsequently, we determined that the minimum IBI of 9 ms must then be used to separate individual burst events.

From each response to the looming stimulus, we were able to extract the DCMD burst duration, number of spikes in each burst, intra-burst-spike-interval (mean ISI in a burst), the peak firing rate within bursts, the IBI (time between the first spike of each burst) and overall percentage of spikes in bursts for all trials. We examined all data relative to the time of collision (TOC) and compared within three epochs of stimulus presentation: (1) the entire stimulus duration; (2) the beginning of object motion (start of stimulus) up to 200 ms before TOC; and (3) 200 ms before TOC up to TOC. This was done to demonstrate how the bursting behavior changed as the object expanded and is supported by observations of our PSTH and previous studies on locusts that showed behavioral responses occurring ~200 ms before object collision (Gray et al., 2001; Matheson et al., 2004).

### Burst Firing Across Varied Stimulus Parameters

To determine if DCMD bursting occurred across varied stimulus parameters, we re-analyzed a subset of data from previous experiments that used the same stimulus size and trajectory (Dick and Gray, 2014). Those experiments used the same setup described here and varied the approach speed (350, 175, 116.7, 87.5 and 75 cm/s) of a 7 cm disc to produce five distinct *l/|v|* values; 10, 20, 30, 40 and 50 ms, respectively. Each approach was presented once to each of 20 locusts. We applied the burst detection algorithm described above and plotted the firing rate of the full response, bursts, spikes in bursts and isolated spikes relative TOC. Gabbiani et al. (1999) showed that DCMD peak firing rates and times are linearly related to *l*/|*v*|. Therefore, to determine if this relation holds for DCMD burst properties, we also plotted the peak and valley firing rates and times against the *l*/|*v*| values. Parameters of linear regression lines through these data are shown in Table 1. We considered *r*^2^ *values* > 0.5 to represent the strongest correlations of the data.

**Table 1:** **Parameters from linear regressions between stimulus expansion properties (*l*/|*v*|) and parameters of full DCMD responses, bursts and isolated spikes**.

Response parameter	Slope	*y*-intercept	*r*^2^
Full response—peak rate	−1.22	236	0.96
Bursts—peak rate	0.01	32	0.02
Spikes in bursts—peak rate	−1.27	236	0.95
Isolated spikes—peak rate	0.03	20	0.32
Isolated spikes—valley rate	0.06	0.33	0.61
Full response—peak time	−3.90	12	0.99
Bursts—peak time	−4.17	−72	0.87
Spikes in bursts—peak time	−3.88	11	0.99
Isolated spikes—peak time	0.86	−499	0.02
Isolated spikes—valley time	−1.99	−47	0.40

*Regressions lines plotted in Figures 7A,B. Gray shaded cells indicate strongest correlations (*r*^2^ > 0.5)*.

### Statistical Analysis

Significance of DCMD firing parameters was assessed at *P* < 0.05. Tests were performed using R (The R Foundation for Statistical Computing) and SigmaStat 3.5 and all data were plotted using SigmaPlot 12.5 (Systat Software Inc., Richmond, CA, USA). Statistical tests used to compare various parameters of the DCMD spike train were a student’s *t*-test (*t*) for parametric data and a Mann-Whitney Rank Sum Test (*U*) for non-parametric data. We compared the homogeneity of slopes of whole responses by using a one-way repeated measures analysis of covariance (*F*, RM ANCOVA) and one-way repeated measures analysis of variance (*F*, RM ANOVA) was used for comparing burst behavior parametrically distributed and a Friedman RM ANOVA on ranks (χ^2^) for non-parametrically distributed data. Data was plotted as column graphs with the mean ± SD or as box plots with medians and 90th and 10th percentiles for parametric and non-parametric data, respectively. Specific tests used are indicated by their respective test statistics in appropriate sections of the results with the degrees of freedom subscripted. Means were reported in text with ± SD, while medians were reported with the 25th and 75th percentiles in parentheses.

## Results

Each response from each animal was processed through the same burst detection analysis and all DCMD responses (600 approaches in total) were grouped, time aligned to TOC, and analyzed in Neuroexplorer. Previous studies have shown that low DCMD firing rates that occur around 200 ms before collision may trigger avoidance steering responses in rigidly tethered locusts (Gray et al., 2001; Matheson et al., 2004). In addition, we observed a higher degree of oscillations in the firing rate approximately 200 ms before TOC (Figure 1). We subsequently compared DCMD responses based on the three epochs described above (Figure 1).

### Consistency of DCMD Responses to Looming

Typical DCMD responses (an increasing spike rate that reaches a peak near TOC; Rind and Simmons, 1992; Gabbiani et al., 1999; Gray et al., 2001; Guest and Gray, 2006) were generated when we presented locusts with looming visual stimuli. Responses were consistent and showed clear oscillations in the firing rate up to 200 ms before TOC, after which the firing rate steadily increased (Figure 1B). To test for any effect of hysteresis we compared the first and last looming stimulus for each animal. There was no significant difference in the time (*t*_38_ = −0.32, *P* > 0.05) and amplitude (*t*_38_ = 0.50, *P* > 0.05) of peak firing rate, peak width at half height (PWHH; *U*_38_ = 252, *P* > 0.05), or total number of spikes (*t*_38_ = 0.61, *P* > 0.05). Therefore, repeated stimulation over the course of the experiment had no effect on DCMD responses to looming.

To test the consistency among all responses, we compared four response parameters that generally describe the DCMD response to looming. The time and amplitude of peak firing and the PWHH were not significantly different among all trials (time: *F*_29_ = 0.78, peak: *F*_29_ = 1.49 PWHH: *F*_29_ = 1.12, *P* > 0.05). The only difference we found was in total spike number (*F*_29_ = 1.80, *P* < 0.05). Thus, all responses were, overall, consistent (Figure 1B). For all presentations from every animal, the peak amplitude was 317 ± 43 spikes/s, time of peak was 50 ± 39 ms before TOC, number of spikes was 52 ± 16, and the PWHH was 28 ± 13 ms. These values are typical of DCMD responses to small disks and based on the properties of our stimulus (*l*/|v*|* = 12 ms; Gray et al., 2001; Guest and Gray, 2006; McMillan and Gray, 2012).

### Evidence of Bursting in ISI Return Maps, ISI Distribution Over Time, ISIh, and Autocorrelations

Examining the ISI distribution of a spike train and determining the frequency of occurrence of the various intervals at which the neuron discharges (plotted in the form of a return map or histogram) provides information about the fundamental firing properties of the neuron. Evidence of oscillatory and bursting activity may also be revealed using a power spectral analysis or autocorrelation method (Israel and Burchiel, 2005). We first plotted an ISI return map for all 600 approaches (Figure 2A) as well as a joint ISI heat map to reveal local density of clusters (Figure 2B). We then generated a time aligned ISI distribution (Figure 2C). For intervals up to 50 ms, the ISI return map illustrated a fan shaped distribution of points with multiple distinct clusters (highlighted in Figure 2A) that were consistent with local density distributions (Figure 2B). A bursting neuron will generally be represented by a triangular ISI return map with three separate clusters of points; clusters of short ISIs along the *x* and *y*-axis represent the ISIs that follow bursts and the first ISIs of each burst, respectively, clusters in the bottom left corner represent intra-burst ISIs and the more scattered clusters represent ISIs between isolated spikes (Szücs, 2007). A non-bursting neuron will not display any clustering at short intervals along the axes (Marsat and Pollack, 2012). Using an ISI of 8 ms to define spikes within putative bursts, we found clusters of ISIs (Figures 2A,B), which is consistent with the distribution from a bursting neuron.

To determine if the ISI distribution was related to the stimulus, we plotted the ISIs over time of stimulus approach, relative to TOC (Figure 2C). We found that while overall the intervals decreased as the stimulus approached TOC, leading up to 200 ms before TOC, there were ISI clusters between 1–10 ms (pink arrows in Figure 2C). If the stimulus was encoded by rate alone, we should expect to only observe an overall decrease in ISIs as the stimulus approached TOC. Clusters were masked by the density of the data from 200 ms before TOC to TOC (gray shaded area in Figure 2C). During this time the ISIs decreased progressively as the object approached. These data suggest that bursting is dynamic across a time in which the stimulus properties change (i.e., the object gets closer) and therefore, subsequent analysis included the three stimulus epochs: (1) the entire stimulus duration; (2) the beginning of object motion (start of stimulus) up to 200 ms before TOC; and (3) 200 ms before TOC up to TOC.

The ISI distribution is a commonly used criterion to determine if burst activity is present in single neurons. A bursting neuron will display a bimodal ISI distribution and a limit value is used to separate isolated spikes from spikes grouped into bursts based on the shape of the ISI distribution (see, for example, Metzner et al., 1998; Gabbiani and Krapp, 2006; Marsat and Pollack, 2006; Oswald et al., 2007). An autocorrelation gives the probability of a spike occurring at different time intervals following each spike (time = 0 ms) in the response. Bimodal peaks within the autocorrelation are present when a neuron generates short ISIs within a burst and longer interburst ISIs (the time between spikes at the start of a burst). Figure 3 plots 100 ms normalized ISIh and autocorrelations, each with a 1 ms bin width, for all three stimulus epochs and event types (full DCMD response, bursts and isolated spikes). For the full DCMD response (Figure 3A) the epoch leading up to 200 ms before TOC yielded a strong bimodal ISI distribution with the most frequent ISIs occurring from 1–8 ms (gray shaded area) and the second most frequent ISI occurring around 40 ms. There was also a less-pronounced bimodal ISI distribution for the response to the entire stimulus and a strong unimodal distribution was present in the time window from 200 ms before TOC to TOC. However, the autocorrelation analysis revealed a strong bimodal distribution for all three stimulus epochs (Figure 3A, right panel), with corresponding peaks from 1–8 ms and around 40–50 ms. We also observed peaks of 40 ms across all stimulus epochs in the ISIh and autocorrelation plots for bursts (Figure 3B) but not for isolated spikes (Figure 3C). These data further support the presence of bursting in response to looming.

### Burst Algorithm Isolates Single Spikes and Bursts

Based on the above results we hypothesized that 30–50 ms may be an IBI and 1–8 ms may be the ISI within each burst (i.e., the intra-burst interval). We developed a burst detection assay in Neuroexplorer (see “Materials and Methods” Section) where all spikes were classified as either isolated spikes or grouped into bursts of two or more spikes with ISIs less than 8 ms (see gray shaded areas in Figure 3). Figure 1A shows an example extracellular recording from a single animal and the results from our burst analysis (gray squares).

### Analysis of the DCMD Bursting Activity

**Figure 4:**
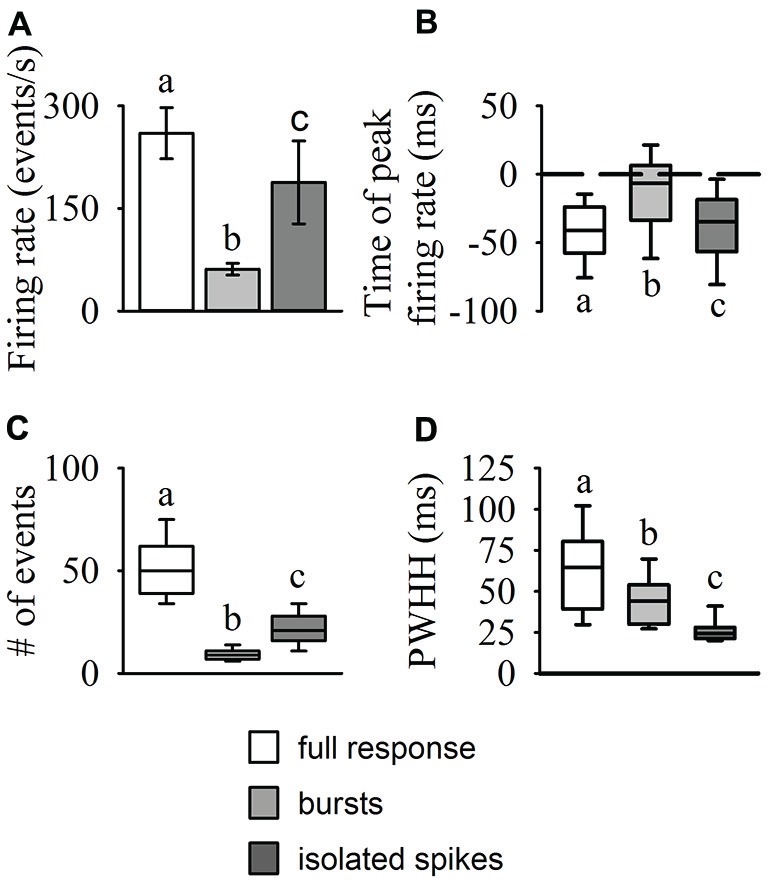
**Statistical comparisons of DCMD response types (full responses, bursts, and isolated spikes).** The amplitude and time of peak firing rate **(A,B)**, total number of events (spikes or bursts), **(C)** and peak width at half height (PWHH); **(D)** were all significantly different between each of the response types. The values reported for the full DCMD response are consistent with previously published work (see “Results” Section). The isolated spike rate generally increased up to 200 ms before TOC, generating a relatively short but high peak firing rate, while bursting generally increased and peaked from 200 ms before TOC to TOC. Different letters above or below bars or boxes represent significant differences between parameters within each panel. Significance assessed at *P* < 0.05.

Results from our burst analysis revealed that both bursts and isolated spikes are related to the stimulus (Figure 1C). Near the end of object approach, where the firing rate is at its peak, our analysis of the entire response placed almost all of the spikes within bursts while isolated single spikes increased up to 200 ms before collision but decreased rapidly soon after (blue line in Figure 1B). Similar to the mean DCMD response (Figure 1B), burst frequency increased as object size increased during approach. Figure 4 summarizes the different response parameters of each category. We found that the DCMD peak firing rate before burst detection (full DCMD response) had the highest firing rate, followed by single spikes and then bursts (*χ^2^_599_* = 1164.69, *P* < 0.001, Figure 4A). The time of peak firing also varied among the response types with the earliest peak occurring with the full DCMD response at 41 (range: 24–58) ms before TOC and latest peak occurring with bursts at 7 (range: 6–33) ms before TOC (*χ*^2^_599_ = 213.28, *P* < 0.001, Figure 4B). While both the full DCMD spike rates and burst rates increased as the stimulus approached TOC (Figure 1B), the histogram representing isolated spikes in Figure 1B (blue line) shows an increase up to 200 ms before TOC, followed by a drop around TOC and a subsequent rise after TOC. Also, note that many of the DCMD burst peaks occurred after TOC (Figures 4B, 1B). We found that the median number of spikes for the full response was 50 (range: 39–62), while the median number of bursts was significantly lower at 9 (range: 7–11), and there were 21 isolated spikes (range: 16–28) per response (*χ*^2^_599_ = 1165.74, *P* < 0.001, Figure 4C). Finally, the shape of each response was significantly different with isolated spiking having the shortest PWHH (*χ*^2^_599_ = 578.59, *P* < 0.001, Figure 4D). In summary, there was a relatively low bursting rate rate around TOC, although bursting still increased in frequency leading up to collision, while single spiking dropped as the stimuli approached TOC.

**Figure 5:**
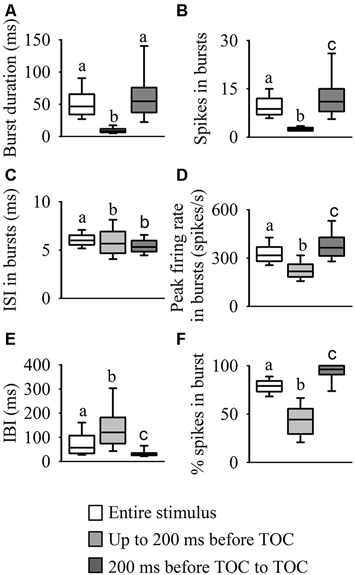
**Statistical comparisons of burst structure.** We performed a burst analysis on three phases of the DCMD response to looming: during the entire stimulus duration (white boxes) and the two stimulus epochs: up to 200 ms before TOC (light gray boxes), and from 200 ms before TOC to TOC (dark gray boxes). Examination of each burst revealed that as the stimulus approached the locust, there was an increase in burst duration **(A)**, spikes in bursts **(B)**, peak firing rate in bursts **(D)** and percentage of total spikes contained in a bursts. **(F)** However, the interval between bursts **(E)** decreased as the stimulus approached TOC and the ISI within each burst **(C)** was similar for all three phases. Different letters above boxes represent significant differences between parameters within each panel. Significance assessed at *P* < 0.05.

Analysis of the internal burst structure (Figure 5) revealed that the median burst duration for the entire stimulus (47 ms, range: 37–66) was similar to the duration in the epoch from 200 ms before TOC to TOC (55 ms, range: 34–76), while the duration of bursts leading up to 200 ms before TOC was much shorter (9 ms, range: 6–12, *χ^2^_599_* = 559.84, *P* < 0.001, Figure 5A). As described above, the highest number of spikes in bursts was present from 200 ms before TOC to TOC (11, range: 8–15), while the number leading up to 200 ms before TOC was lower (3, range: 2–3) and during the entire stimulus there was a median of 9 (range: 7–12, *χ*^2^_599_ = 610.17, *P* < 0.001, Figure 5B). Although the ISIs within bursts throughout the stimulus presentation appeared relatively constant (~6 ms across all three epochs), the median ISI in each burst was significantly higher for the entire stimulus (*χ*^2^_599_ = 192.57, *P* < 0.001, Figure 5C). The epoch from 200 ms before TOC to TOC had the highest peak firing rate in each burst at 365 spikes/s (range: 313–427) vs 317 spikes/s (range: 280–369) and 217 spikes/s (range: 183–261) up to 200 ms before TOC and the entire stimulus, respectively (*χ*^2^_599_ = 545.71, *P* < 0.001, Figure 5D). The median interburst-interval (IBI) during the stimulus epoch from 200 ms before TOC to TOC was significantly lower than the other two stages at 30 ms (range: 25–35) vs 120 ms (range: 75–183) and 57 ms (range: 34–107) for up to 200 ms before TOC and the entire stimulus, respectively (*χ*^2^_599_ = 263.273, *P* < 0.001, Figure 5E). While 80% (range: 73–84) of spikes were in bursts during the entire stimulus presentation, almost all spikes were present in bursts from 200 ms before TOC to TOC at 96% (range: 91–100) and only 44% (range: 30–56) of spikes were in bursts leading up to 200 ms before TOC (*χ*^2^_599_ = 621.25, *P* < 0.001, Figure 5F).

Overall, the IBI distribution histogram and autocorrelation analysis for the entire stimulus and from 200 ms before TOC to the end of stimulus was slightly bimodal, with one peak around 18 ms and a second more pronounced peak around 40 ms (Figure 3B); the median IBIs for these two phases was 30 ms and 57 ms, respectively (Figure 5E). While responses during the phase leading up to 200 ms before TOC had a more unimodal distribution, the peaks in both the IBIh and burst autocorrelation was around 50 ms (with a median of 120 ms). The distribution of single spikes was relatively unimodal for the ISIh and there was no clear trend in the autocorrelation analysis (Figure 3C). This unimodal distribution (without oscillations) of single spikes relates to its increasing firing rate in response to the looming stimuli.

These analyses revealed important new findings about DCMD responses to looming. First, our algorithm was capable of extracting bursts even during the typical high frequency firing pattern near TOC and these bursts appeared to dominate leading up to TOC (where almost all spikes are within bursts). Second, two modes of firing appeared in the DCMD response (isolated spikes and bursts) and the switch from one to another occurred at a behaviorally-relevant time during an approach.

### Relation between Burst Firing Properties and Stimulus Expansion Parameters

**Figure 6:**
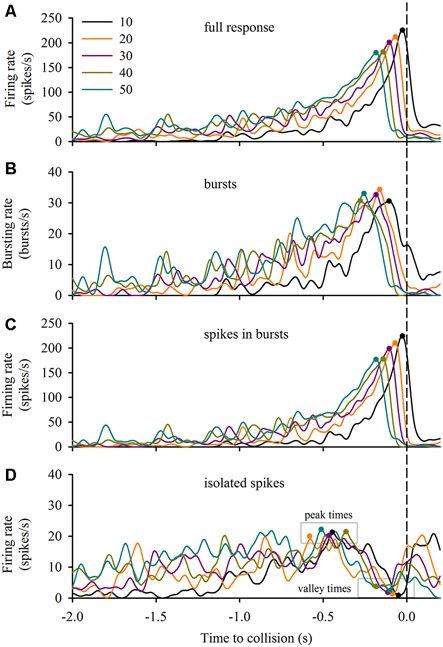
**Bursting in response to varied stimulus expansion parameters.** We applied our bursting algorithm to a subset of data from Dick and Gray (2014) and plotted the mean firing rate (*n* = 20 locusts, see “Materials and Methods” Section) of the full response **(A)**, bursts **(B)**, spikes in bursts **(C)** and isolated spikes **(D)** relative to the time of collision (TOC; dashed vertical line). The inset in **(A)** identifies the *l/|v|* value of each plot in each panel. Filled circles in each plot represent the time of the peak or valley.

**Figure 7:**
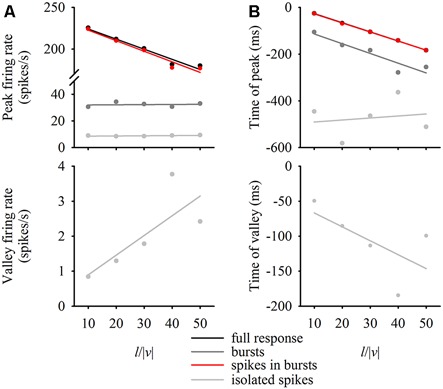
**Relationship between expansion parameters and firing properties.** For each *l/|v|* value, we plotted the firing rate at the peak of the full response, bursts, spikes in bursts and isolated spikes (**A**, top panel) as well as the firing rate at the valley of the isolated spikes (**A**, bottom panel). We also plotted the time of the relative peaks (**B**, top panel) or valleys (**B**, bottom panel) against *l/|v|*. Data represent mean values from 20 locusts and were fit with linear regression lines (see Table 1 for regression parameters).

DCMD responses to a looming disc varied across different approach velocities, and subsequent expansion properties. For objects with lower *l*/|*v*| values (faster approach velocities) the full DCMD response reached a higher peak firing rate later in the response (Figure 6A). After separating bursts and isolated spikes, we found that bursts (Figure 6B) and spikes in bursts (Figure 6C) followed a similar pattern whereas the firing rate of isolated spikes reached a less pronounced peak and then declined to a valley before time of collision (Figure 6D). Moreover, the rate of isolated spikes decreased at the time that the rate of bursts and spikes in bursts increased, similar to the results from the data in this study (Figure 6B). Figure 7A and Table 1 show that the peak firing rate of the full DCMD response and spikes in bursts were strongly inversely correlated to *l*/|*v*|, the firing rate at the valley of isolated spikes was positively correlated to *l*/|*v*|, and that the peak bursting rate (32 ± 1.6 bursts/s, mean ± SD) and isolated spike peak rate (21 ± 0.9 spikes/s) was invariant to approach velocity. The time of peak firing, however, was strongly negatively correlated to *l*/|*v*| for the full response, bursts and spikes in bursts (Figure 7B, Table 1). While the time of the valley of isolated spikes was weakly negatively correlated with *l*/|*v*|, the peak rate of isolated spikes was invariant to approach velocity. Note in the upper panels in Figures 7A,B that the plots and regressions of the full DCMD response (black) and spikes in bursts (red) are nearly indistinguishable. We also observed that for each *l*/|*v*| value, the peak of burst firing occurred 92 ± 26 ms earlier than the full DCMD peak response and that this difference was invariant to approach velocity (*y* = −0.27, *x* – 84, *r*^2^ = 0.03).

## Discussion

Given that forewing asymmetry generates avoidance behaviors during flight (Robertson and Reye, 1992; McMillan et al., 2013) and that DCMD activity may gate information regarding an impeding collision into post-synaptic motor neurons that control the forewings (Simmons, 1980; Reichert et al., 1985; Santer et al., 2006), it is likely, as suggested by previous studies (Simmons and Rind, 1992; Judge and Rind, 1997; Gabbiani et al., 1999; Gray et al., 2001; Santer et al., 2006) that the LGMD/DCMD pathway is important for collision avoidance behavior during flight. However, to date, no experiments have rigorously tested the possibility of DCMD bursting, as bursting is recognized as an important component of sensory processing in many systems and can have important consequences for coding (Lisman, 1997; Brenner et al., 2000). An important finding from Gabbiani and Krapp (2006) was that although positive currents drive the LGMD to burst, a large number of spikes from larger depolarizing currents obscure the bursting activity. Therefore, evidence of bursting in previously described LGMD/DCMD responses to looming visual stimuli may have been obscured by an exceptionally robust stimulus. Moreover, the differences in stimulus types presented (i.e., context-dependent responses) and variation between and within individuals may have hindered the detection of DCMD bursts in other studies. Evidence that sensory neurons encode behaviorally relevant stimuli using bursts has been proposed for many other systems (Guido et al., 1995; Lesica and Stanley, 2004; Oswald et al., 2004; Marsat and Pollack, 2006; Eyherabide et al., 2008; Sabourin and Pollack, 2009), but had not been investigated or quantified rigorously in the locust DCMD. Observations of Gaussian smoothed DCMD firing rate responses to lateral looming stimuli show repetitive patterns of spiking that may represent a bursting pattern. Our results suggest the presence of a temporal code, in the form of bursts, within the DCMD responses we quantified. Moreover, we found that there may be behavioral implications to the timing of these bursts, given that they occurred at frequencies that are consistent with frequencies generated by the locust’s forewings during tethered and loosely tethered flight (20–25 Hz, Robertson and Johnson, 1993; McMillan et al., 2013). We also found that burst frequency also increased with edge expansion during an approach (Figure 1B). Therefore, it is likely that DCMD bursts encode dynamic aspects of the visual stimulus.

While further experiments are needed to test for a direct relationship between bursting and the output of associated flight muscle motor neurons, the data presented here suggest that bursting may be a way of gating information into downstream flight circuitry (Santer et al., 2005). Bursting in the DCMD may provide the encoding link in gating information into postsynaptic motor neurons or the flight central pattern generator (CPG). Santer et al. (2006) found that high frequency DCMD spikes caused summation of EPSPs in a forewing elevator motor neuron (Mn84) and occurred with the onset of the Mn84 burst that elevates the forewings into a gliding posture. Avoidance responses in loosely tethered flying locusts are initiated within a single wing beat (<40 ms), where the presence of an additional spike from the forewing depressor muscle on the inside of the turn, is enough to initiate the generation of an asymmetry that within a few hundred milliseconds, generates an avoidance turn (McMillan et al., 2013). Conceivably, spikes from the DCMD, if gated into the flight rhythm, could be enough to initiate forewing depression (whether it be the left or right depressor muscle).

The presence of many spikes within a burst may also be related to the reliability of the signal; which in the case of the DCMD may represent whether or not the locust can predict when an object will collide with it. If a high frequency burst of spikes were transferred into the motor neurons (such as was proposed by Santer et al., 2005), spike redundancy would be advantageous, especially considering the possibility of synaptic failure and the potential for bursting to improve the coding of salient sensory cues (Lisman, 1997; Brenner et al., 2000). Indeed, the variability in spike firing times exists in many neurons and even if variability in spiking may not be part of a sensory signal, it may be an important part of the accurate processing of the signal (Stein et al., 2005). Variability in the timing of bursts means that each burst may arrive at different times during the phases of each wing beat, consequently generating a slightly different behavioral response. Although DCMD responses alone may not reliably predict the behavior of the locust, variability in behavioral responses to similar visual stimuli may allow the locust to remain elusive if being attacked by an aerial predator (Santer et al., 2006; McMillan et al., 2013). Therefore, bursting may aid in the transfer of visual information, effectively gating an underlying rate code into the rhythmical flight circuitry.

Although results from our ISI distributions (Figure 2) and histograms (Figure 3) suggest that while rate coding alone may not characterize DCMD responses to simple looming stimuli, the presence of isolated spikes along with bursts implies that two codes may be present, which are expressed depending on stimulus dynamics. Other sensory neurons involved in generating avoidance behaviors may use two modes of encoding in a context dependent manner. For example, the AN2 neuron in crickets may encode threatening bat cries using bursting, while single spiking may encode non-threatening calls from conspecifics (Marsat and Pollack, 2006). After 200 ms before TOC, the DCMD response is characterized by a rapid increase in burst frequency and decrease in isolated spiking. Considering the latency between visual input (for an *l*/|*v|* = 20 ms looming disc) and onset of behavior (~60 ms, see Robert and Rowell, 1992) and the time when flight steering maneuvers are initiated (~180 ms before TOC, Robertson and Johnson, 1993; Matheson et al., 2004), DCMD spikes involved in initiating an intentional steering maneuver must occur ~200 ms before TOC (Robertson and Johnson, 1993; Matheson et al., 2004; Gray, 2005); if the DCMD continues to fire after 200 ms before TOC, an evasive glide may occur (Santer et al., 2006). In many nocturnally flying insects, such as moths, high frequency spiking occurs when an attacking bat is near and similar last chance avoidance behaviors are performed (Triblehorn and Yager, 2005). We found that single spiking increases up to and decreases after 200 ms before TOC, whereas burst frequency gradually increases up to 200 ms before TOC followed by a rapid increase to TOC. It is possible that low burst rates (and relatively high single spikes rates) before 200 ms may elicit responses that result in subtle trajectory changes, while the high frequency bursting (and low single spike frequency) near the TOC may result in an evasive glide (Santer et al., 2006). These predictions need to be tested with looming visual stimuli representing, for example, another conspecific in a swarm (non-threatening and non-colliding objects with relatively low visual impact) and an approaching predator (threatening with relatively a large visual impact). Indeed, the identification of DCMD bursting in response to simple looming will now lead the way to further experiments designed to address specific questions regarding coding of complex visual information. Data from these experiments will be incorporated into an evolving model of DCMD responses to stimuli that travel along multiple trajectories (see Silva et al., 2015).

Re-analysis of a subset of existing data (Dick and Gray, 2014) provided a first step in understanding a putative role for bursting in coding visual motion. The data suggest that much of the information of a looming object is carried in the form of bursts, as demonstrated by a near identical full response and response of spikes in bursts across different approach velocities (Figures 6, 7). Previous work has shown that multiplexing of DCMD firing properties (firing rate threshold, peak firing time and spike count) may be important for triggering jump responses to looming objects (Fotowat et al., 2011). Our findings here suggest that multiplexing may also exist such that a constant burst rate that could detect an approach at behaviorally-relevant time (see McMillan et al., 2013), irrespective of approach speed, whereas the firing rate of spikes within bursts could be encoding approach velocity. The peak rate and time of isolated spikes is relatively invariant to approach velocity whereas the firing rate and time of the isolated spike valleys are positively and negatively correlated, respectively, to *l/|v|*. These isolated spike correlations may be a result of spikes clumping into bursts as the object gets closer. These findings provide motivation for future experiments to explore the possibility of multiplexing across of range of complex stimulus parameters.

Understanding the nature of the information that postsynaptic motor neurons extract from the DCMD spike train during a looming presentation is required to elucidate the role of this visual pathway in collision avoidance. Although burst encoded sensory stimuli have been linked to behavioral roles in other insects (Marsat and Pollack, 2006), it is necessary to establish a link in the timing of DCMD bursts in a behavioral context. Understanding the downstream role of sensory neurons in a behavioral context is imperative to interpret a sensory signal, as the timing of a spike to some internal event may be considered unreliable if the observer is not aware of such an event (VanRullen et al., 2005). A clearer understanding of the role of sensory neurons, such as the LGMD/DCMDs, in affecting post-synaptic neurons and the role of those affected neurons in generating behaviors will help reveal a reliable sensory code. Future experiments using simultaneous DCMD and muscle recordings during flight will uncover the nature of the information conveyed by this visual pathway and provide further insights into general principles of sensory coding and information transfer.

## Conflict of Interest Statement

The authors declare that the research was conducted in the absence of any commercial or financial relationships that could be construed as a potential conflict of interest.
